# Three-Dimensional Imaging of *Prox1*-EGFP Transgenic Mouse Gonads Reveals Divergent Modes of Lymphangiogenesis in the Testis and Ovary

**DOI:** 10.1371/journal.pone.0052620

**Published:** 2012-12-20

**Authors:** Terje Svingen, Mathias François, Dagmar Wilhelm, Peter Koopman

**Affiliations:** Division of Molecular Genetics and Development, Institute for Molecular Bioscience, The University of Queensland, Brisbane, QLD, Australia; Instituto Gulbenkian de Ciência, Portugal

## Abstract

The lymphatic vasculature forms a specialized part of the circulatory system, being essential for maintaining tissue fluid homeostasis and for transport of hormones, macromolecules, and immune cells. Although lymphatic vessels are assumed to play an important role in most tissues, their morphogenesis and function in the gonads remains poorly understood. Here we have exploited a lymphatic-specific *Prox1*-EGFP reporter mouse model and optical projection tomography technology to characterize both the temporal and spatial development of the lymphatic vessel network in mouse testes and ovaries. We find that lymphangiogenesis in the testis is initiated during late gestation, but in contrast to other organs, lymphatic vessels remain confined to the testis cap and, unlike blood vessels, do not infiltrate the entire organ. Conversely, lymphatic vessels invade the ovarian tissue, beginning postnatally, and sprouting from preexisting lymphatic vessels at the extraovarian rete. The ovary develops a rich network of lymphatic vessels, extending from the medulla into the surrounding cortex adjacent to developing follicles. This study reveals distinct patterns of lymphangiogenesis in the testes and ovaries and will serve as the basis for the identification of the divergent molecular pathways that control morphogenesis and the function of the lymphatic vasculature in these two organs.

## Introduction

The lymphatic vasculature consists of an extensive network of small, blind-ended capillaries, larger vessels and collecting ducts [1]. It drains extracellular fluids from tissues and organs to maintain tissue homeostasis, and transports lipids, hormones, white blood cells and antigen-presenting cells to lymphoid organs [1], [2], [3]. The lymphatic vasculature is thus integral to normal physiological functioning of the organism and acts as a defense mechanism against infections and disease.

The lymphatic vasculature originates from the anterior cardinal vein during embryonic development; around 9.5 days post coitum (dpc) in the mouse. Initially, a subset of venous endothelial cells expresses two transcription factors, SRY-box containing gene 18 (SOX18) and the nuclear receptor COUP-TFII (also named NR2F2; nuclear receptor subfamily 2, group F, member 2), which directly induce the expression of Prospero-related homeobox 1 (PROX1), a factor crucial for lymphatic development [4], [5], [6]. These regionally defined cells, also expressing Lymphatic vessel endothelial hyaluronan receptor-1 (LYVE1), undergo a combination of morphogenetic changes that generate lymph sacs and streams of migrating cells to establish the initial lymphatic plexus [7]. Most other tissues subsequently acquire a lymphatic vasculature at various stages during development through lymphangiogenesis – the formation of new lymphatic vessels from pre-existing vessels - a process largely dependent upon PROX1 function [8].

In mammals, testes and ovaries are also connected to the lymphatic network, and it is assumed that, as in other tissues, lymphatic vessels drain extracellular fluids and macromolecules from the gonadal interstitium to support normal physiology and function. Lymphatics of mammalian gonads were recognized more than 40 years ago, and have since been characterized in diverse animal species including primates, bull, goat, pig, rabbit and rodents [9], [10], [11], [12], [13], [14], [15]. However, these studies typically focused on testes and ovaries from adult animals, and lymphangiogenesis during gonad development remains largely unexplored.

In mice, superficial lymphatic vessels have been identified in both testes and ovaries during late gestation, around 17 days *post coitum* (dpc), where they presumably grow from a pre-existing network of vessels at the gonad-mesonephros border [16]. In contrast, blood vascularization occurs much earlier in the developing mouse gonads [16], [17], and hence there appears to be a significant delay in lymphangiogenesis in both testes and ovaries. Also, the lymphatic network in fetal mouse gonads has been examined only in whole-mount tissues, such that no information exists regarding a putative lymphatic vascular network with the gonadal interstitium early in fetal life.

It is important to consider reported differences between species when analyzing the lymphatic vasculature of the gonads. A recent study suggested that the adult testis of smaller rodents does not possess lymphatic vessels within the interstitial space and that the vessels are restricted to the testis cap; the tunica albuginea [18]. In contrast, early studies on larger mammals have reported lymphatics in the interstitial space between seminiferous tubules [10], [11]. With regards to ovaries, however, there appear to be no significant differences in the spatial distribution of the lymphatic vasculature between larger mammals and smaller rodents. Here, the lymphatic vessels typically localize to the peripheral zone of the corpus luteum and to the thecal layer surrounding growing follicles, likely merging in the medulla and rete ovarii [12], [14], [15], [19], [20], [21].

Although there appeared to be no major differences in ovarian lymphatics between mammalian species, emerging evidence strongly suggests sex-specific differences both with regards to lymphangiogenic initiation and vessel distribution within the gonads. Superficial lymphatic vessels of the ovary were suggested during fetal life [16], however, more recent studies implied that ovaries are devoid of lymphatic vessels until after birth and that lymphatic vessels invade the prepubertal ovary via the mesovarium into the medullary region from around 10 days *post natum* (dpn) [21]. One potential explanation for this discrepancy is the use of different molecular markers for lymphatic vessels. Also, the fetal ovary is surrounded by epithelial tissue, which may itself possess a network of lymphatic vessels not readily separable from the ovary proper. Regardless, the initiation of lymphatic development of both testes and ovaries remains unclear and warrants further analyses.

The visualization of lymphatic development has commonly relied on markers such as LYVE1 and podoplanin (PDPN). However, these proteins are also expressed by cell types other than lymphatic endothelial cells, including embryonic venous endothelial cells [22], high endothelial venules [23], and granulosa cells of the developing ovarian follicles [21], [24]. Moreover, neither is expressed uniformly across different sub-types of lymphatic vessels [25]. On the other hand, *Prox1* is regarded as a hallmark of lymphangiogenesis [6], [26] and has served as a reliable biomarker of lymphatic vessel formation and maintenance [8], [25] despite also being essential for venous valve formation [27].

In this study, we took advantage of a *Prox1*-EGFP reporter mouse (Tg(*Prox1*-EGFP)221Gsat/Mmcd) that faithfully recapitulates endogenous PROX1 expression [7], [28] to further visualize gonadal lymphangiogenesis. By analysing transgenic gonads using conventional fluorescence microscopy, confocal microscopy and optical projection tomography (OPT), we captured the spatiotemporal patterning of the lymphatic network at a level of detail not previously possible. These experiments offer new insights into both the study of gonad organogenesis and lymphangiogenesis, and map the sex-specific development and distribution of the lymphatic vasculature of the testes and ovaries.

## Materials and Methods

### Animals

*Prox1*-GFP BAC transgenic mice (Tg(*Prox1*-EGFP)221Gsat/Mmcd) were provided by Dr Y.K. Hong (USC, USA) [28]. For this study, homozygous Tg(*Prox1*-GFP) males were mated with wild-type females, both on an outbred CD1 background to create heterozygous Tg(*Prox1*-GFP) offspring for analyses. Non-transgenic offspring were collected from wild-type CD1 matings. Developmental stage was assigned as noon of the day on which the mating plug was observed corresponding to 0.5 dpc. Gonadal sex was determined by morphological assessment. Protocols and use of animals in the described experiments were approved by the Animal Ethics Committee of the University of Queensland, in accordance with the Queensland Animal Care and Protection Act (2001).

### Immunofluorescence

Whole-mount immunofluorescence (IF) imaging of *Prox1*-EGFP was performed on freshly dissected (untreated) tissues in phosphate buffered saline (PBS) or by confocal microscopy following dehydration of PFA-fixed samples in methanol and clearing in benzyl alcohol:benzyl benzoate as described for optical projection tomography. Section IF experiments were performed on 7 µm sagittal sections of paraffin-embedded tissues pre-fixed in 4% paraformaldehyde (PFA) in PBS as previously described [29]. In brief, tissue-sections were dewaxed and rehydrated before treated with Antigen Unmasking Solution (Vector Laboratories, USA), pre-blocked with 10% heat-inactivated horse serum in 0.1% Triton-X in PBS (PBTX), and then incubated with antibodies in blocking solution overnight at 4°C. Sample were washed in PBTX and incubated with secondary antibodies for 2 h, counterstained with 4,6-diamidino-2-phenylindole (DAPI, Sigma) and mounted with 60% glycerol. Images of freshly dissected samples from postnatal *Prox1*-EGFP mice, as well as fetal gonadal samples represented inFig. S1, were acquired using an Olympus BX-51 fluorescence microscope. All other images were acquired on a Biorad Radiance 2100 confocal microscope equipped with three lasers (480 nm Argon ion, 543 nm Green HeNe and 637 nm Red Diode), attached to an Olympus IX70 inverted microscope. Adobe Photoshop was used for subsequent image processing.

### Optical Projection Tomography

Pre-fixed ovaries (4% PFA in PBS) from adult *Prox1*-EGFP mice were subjected to whole-mount IF as previously described [7]. Stained samples were embedded in warm 1% low-melting-point agarose and left until set, adjusting orientation as necessary. Set agarose blocks were glued to aluminium-magnetic mounts. Specimens were then dehydrated in 50% methanol for 18 h with 3 graduated changes of methanol to 100%, and then cleared overnight in benzyl alcohol: benzyl benzoate mixed at a ratio of 1∶2. Once cleared, samples were imaged in a Bioptonics 3001 Optical Projection Tomography (OPT) scanner (Bioptonics, UK). Images were acquired at 0.9° intervals and reconstructed. Stacks were rendered for presentation using IMARIS software.

### Antibodies

Primary antibodies used were: rabbit anti-DDX4 (1∶200, Abcam), rabbit anti-LYVE1 (1∶250; Fitzgerald Industries), chicken anti-GFP (1∶300; Abcam), rabbit anti-HSD3B1 (1∶200; [30]), rabbit anti-PROX1 (1∶500; Angiobio), rat anti-ENG/CD105 (1∶200; BD Biosciences), goat anti-NRP2 (1∶500; R&D Systems), mouse anti-ACTA2 (1∶300; Sigma Aldrich), rabbit anti-FOXL2 (1∶200; [31]) and rabbit anti-CYP11A1/SCC; (1∶200). Anti-CYP11A1 was produced by immunizing rabbits using the following peptides: NKFDPTRWLEKSQNC and SSPRSFNEIPSPGDC (GenScript corporation), affinity purified and tested for specificity. All secondary antibodies were used at 1∶200 dilutions for section IF and 1∶500 for whole-mount IF and were: anti-chicken Alexa 488, anti-rabbit, -rat, -mouse, and -goat Alexa 596, and anti-rabbit Alexa 647 (Molecular Probes/Invitrogen).

## Results

### Testicular Lymphangiogenesis Begins during Late Gestation

To characterize the onset of lymphatic vessel growth in the developing testes, we visualized EGFP expression in XY gonad-mesonephros complexes of *Prox1*-EGFP fetuses from 12.5 dpc onwards. Gonad-mesonephros complex pairs of at least one litter of no less than five embryos of each sex at each developmental stage were analysed by fluorescent microscopy. Representative XY samples between 15.5 and 19.5 dpc are shown in Fig. S1. In summary, no *Prox1*-driven EGFP expression was detected before 14.5 dpc (data not shown), after which weak EGFP signal was detected in the mesonephros. At 16.5 dpc, strong EGFP signal was visible along the spermatic cord, likely following the pampiniform plexus of veins before ending at the rete testis with few vessels sprouting laterally. At 17.5 dpc, extensive branching of superficial EGFP-positive vessels was observed, originating from the spermatic cord/rete testis before fanning out over the testis cap. At 18.5 dpc, the superficial EGFP-positive vessels were spread across the testis surface. However, at this stage it was more difficult to visualize the vessels due to increasing EGFP signal from the testis soma, apparently from within the testis cords.

**Figure 1:**
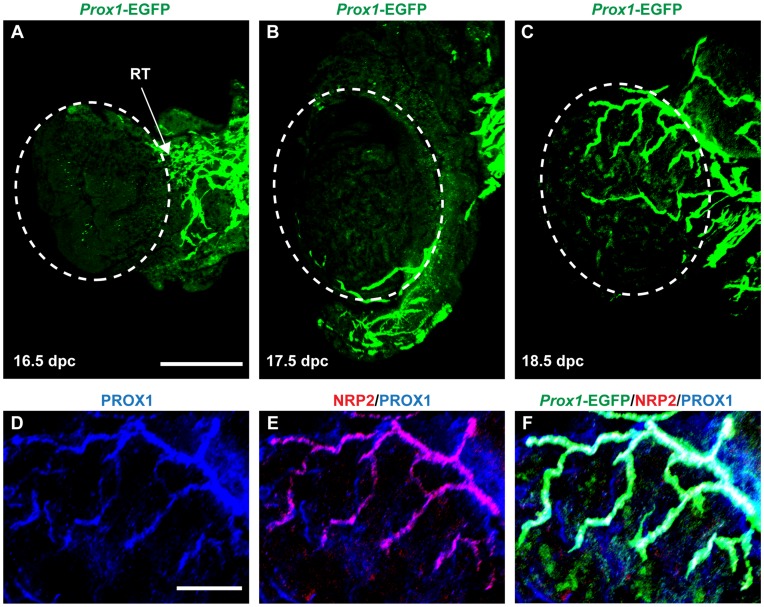
Testis lymphangiogenesis is initiated during late gestation in mice, as visualized by confocal microscopy of whole-mount *Prox1*-EGFP gonad-mesonephros complexes. **A)** At 16.5 dpc, an extensive lymphatic network is present in the adjacent mesonephros, but excluded from the testis. **B)** Lymphatic vessels first appear on the testis surface from 17.5 dpc, seemingly by continuous growth of the vessels already present in the mesonephros. **C)** At 18.5 dpc, lymphatic vessels continue to sprout across the surface of the testis. **D)** Prominent *Prox1*-EGFP positive vessels of the 18.5 dpc testis also express endogenous PROX1, **E)** and the lymphatic marker NRP2, **F)** both overlapping with *Prox1*-EGFP expression. RT = rete testis; scale bars: A = 500 µm, D = 200 µm.

To verify the spatio-temporal *Prox1*-EGFP expression in the developing testis and that EGFP-positive vessels are lymphatic, we performed whole-mount IF on *Prox1*-EGFP gonad-mesonephros complexes from 16.5, 17.5 and 18.5 dpc fetuses, using antibodies to Neuropilin 2 (NRP2) which is expressed in lymphatic vessels [32], and endogenous PROX1 (Fig. 1). At 16.5 dpc, a significant network of EGFP-positive vessels was observed in the mesonephric region, but excluded from the testis proper (Fig. 1A). The development of EGFP-positive vessels across the testis surface from 17.5 dpc observed on fresh *Prox1*-EGFP gonadal samples was confirmed by confocal microscopy (Fig. 1B–C). Also, strong co-localization of endogenous PROX1 (Fig. 1D) and a second lymphatic marker NRP2 (Fig. 1E) with *Prox1*-EGFP in the vessels observed on the testis surface at 18.5 dpc (Fig. 1F) confirmed that *Prox1*-EGFP mimics endogenous PROX1 expression in NRP2-positive lymphatic vessels.

### Lymphatic Vessels of the Testes are Restricted to the Testis Cap

**Figure 2:**
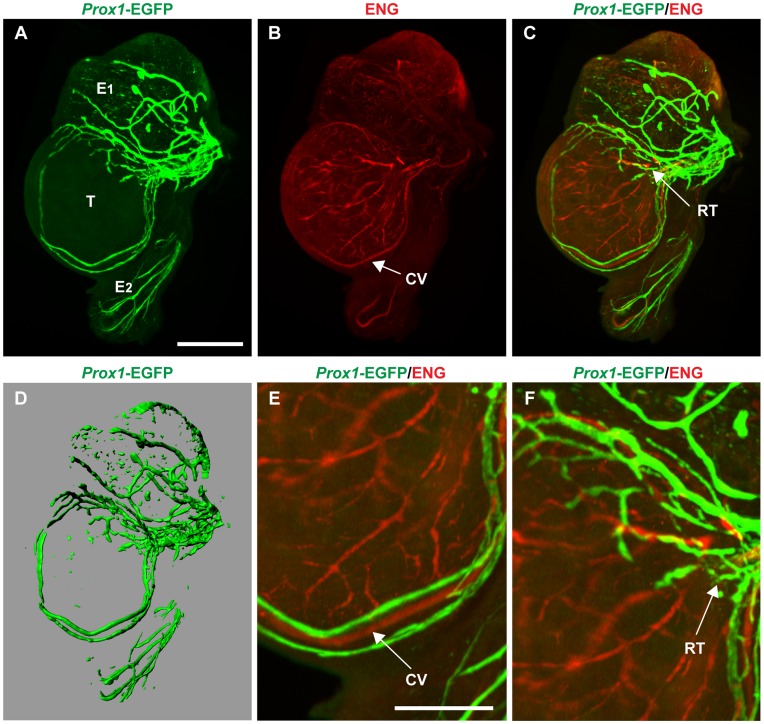
Lymphatic vessels sprout across, but not beyond, the testis cap at 17.5 dpc. Representative images captured from optical projection tomograph, also represented in Vid S1. **A)** During late gestation, EGFP-positive lymphatic vessels are seen growing from pre-existing vessels in the spermatic cord before sprouting across the testis surface. B**)** The fetal testis also contains a rich network of blood vessels visualised by ENG staining, but **C)** the EGFP-positive vessels do not overlap with the more extensive blood vasculature. Yellow areas correspond to lymphatic vessels (green) and blood vessels (red) in different planes. **D)** 3-D model of *Prox1*-EGFP positive lymphatic network during initial development. **E)** Magnified region showing two EGFP-positive lymphatic vessels running parallel to the coelomic (arterial) vessel and **F)** magnified region of the rete testis. T = testis; E1 = head of epididymis; E2 = tail of epididymis; CV = coelomic vessel; RT = rete testis; scale bars: A = 500 µm, B = 250 µm.

To better appreciate the spatial distribution of the lymphatic vasculature, we performed whole-mount IF and OPT analyses on *Prox1*-EGFP transgenic testes at 17.5 dpc. These experiments revealed a strong lymphatic plexus at the mesonephric-gonadal junction with numerous vessels radiating out over the testis cap but never invading the testicular interstitium (Fig. 2 and Video S1).

At 17.5 dpc, the testis and adjoining epididymis already contain a rich network of blood vessels marked by endoglin (ENG) expression (Fig. 2B, C). At this stage, *Prox1*-EGFP-positive lymphatic vessels were observed to span from the pre-existing lymphatic network along the spermatic cord before fanning out over the testis cap (Fig. 2A, C, D). Prominent lymphatic vessels were also observed adjacent to the main testis artery, the coelomic vessel (Fig. 2E, arrow), originating from the rete testis (Fig. 2C, F). Notably no lymphatic vessels were observed to penetrate the tunica albuginea into the interstitial space (Video S1).

**Figure 3:**
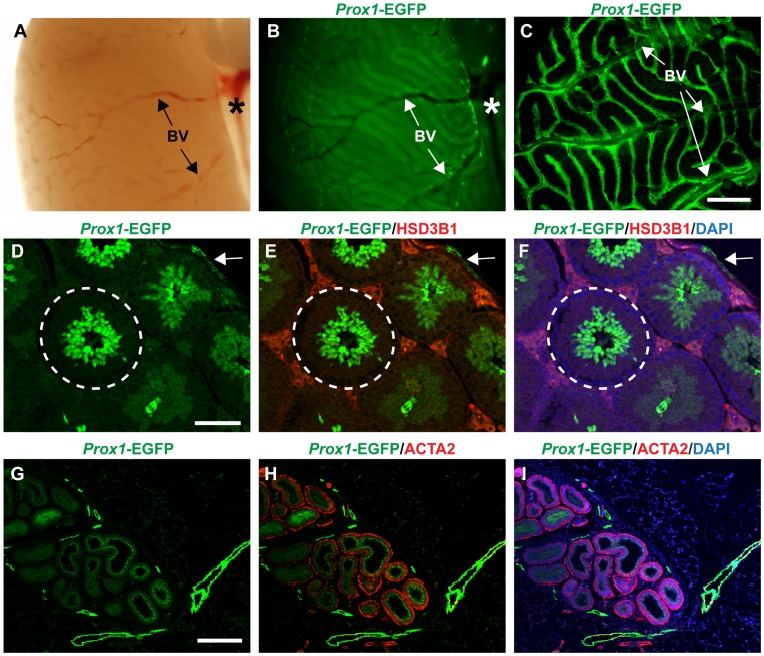
Lymphatic vessels are limited to the tunica albuginea in adult testis. **A–C)** EGFP-positive vessels are readily observed across the surface of the adult testis emanating from the spermatic cord (asterisk). Strong EGFP expression is also observed within the seminiferous tubules. **D–F)** Sectioned *Prox1*-EGFP transgenic adult testis co-stained with the Leydig cell marker HSD3B1 and counterstained with DAPI revealed no lymphatic vessels inside the testis, but within the tunica albuginea (arrow). Additional EGFP expression was verified within the seminiferous tubules (encircled) and localised to spermatids closer to the lumen. **G–I)** Sectioned *Prox1*-EGFP adult epididymis co-stained with the smooth muscle cell marker ACTA2 to demarcate the vas deferens and counterstained with DAPI showed prominent EGFP-positive lymphatic vessels, but also EGFP-positive sperm cells. BV = blood vessel; scale bars C = 600 µm, D = 100 µm, G = 300 µm.

We next analysed adult *Prox1*-EGFP testes. Using whole-mount microscopy, we observed a dense network of lymphatic vessels on the testis surface emanating from the spermatic cord (Fig. 3A–C), however the lymphatic vessels on the testis surface were difficult to visualize due to strong EGFP signal from within the testis (Fig. 3B). Using confocal microscopy, we were able to better visualize the network of EGFP-positive vessels; superficial EGFP-positive vessels were observed over the entire surface of the testis, often associated closely with blood vessels. In contrast, we never observed EGFP-positive vessels in the testis interstitium in adult testis by section IF. Double immunofluorescence of *Prox1*-EGFP testis with the Leydig cell marker HSD3B1 (hydroxyl-delta-5-steroid dehydrogenase, 3 beta- and steroid delta-isomerase 1) detected lymphatic vessels only in the tunica albuginea (Fig. 3D–F; arrow).

Interestingly, *Prox1*-EGFP was also expressed within seminiferous tubules, more precisely in spermatic cells located closest to the lumen (Fig. 3D–F). This *Prox1*-EGFP expression within the spermatic lineage is most likely responsible for the background signal observed from 18.5 dpc in the developing testis (Fig. 1). Further characterization of *Prox1*-specific expression within maturing sperm was not undertaken.

Finally, we found that *Prox1*-EGFP transgene expression marked a lymphatic vascular network within the epididymis, as previously suggested by LYVE1 expression [33]. Again, some *Prox1*-EGFP expression was visible in sperm cells within the vas deferens, outlined by alpha-2 smooth muscle actin (ACTA2) expression (Fig. 3G–I), but was not investigated further in this study.

### Fetal Ovaries are Devoid of Lymphatic Vessels

To characterize the onset of lymphatic vessel growth in the developing ovaries, gonad-mesonephros complexes of *Prox1*-EGFP XX fetuses from 12.5 dpc onwards were visualized by fluorescence microscopy, as shown inFig. S1B. In summary, very few EGFP-positive cells were detected until 15.5 dpc (data not shown). From 15.5 dpc, a strong signal was detected along the Müllerian duct, distal to the ovary. At 16.5 dpc, Müllerian duct-associated EGFP expression extended to the future fimbriae of the oviducts, with additional vessels in the mesonephros (mesovarium) likely following the pampiniform plexus of veins, as in XY embryos, ending close to the extraovarian rete. From 17.5 dpc, no EGFP signal was detected in the uterus proper, but prominent staining of vessels localized to the broad ligament adjacent to the uterus and near the ovary. No superficial EGFP-positive vessels were observed in the ovary during fetal development.

To exclude the possibility of EGFP-positive cells and/or microvessels arising inside the ovary proper during fetal life, we next analysed *Prox1*-EGFP ovary sections at 19.5 dpc. Double-IF using an antibody to the ovary-specific somatic cell marker FOXL2 (Forkhead box L2) revealed pronounced lymphatic vessels in the extraovarian rete, but no EGFP-positive vessels within the ovary proper (Fig. S1C; arrow). XX germ cells in the ovarian cortex were observed to express some *Prox1*-EGFP (Fig. S1C; asterisks).

### Ovarian Lymphangiogenesis Begins Postnatally, Prior to Puberty

It has been suggested that postnatal ovaries possess an internal lymphatic network only from around 10 dpn onwards [21], in contrast to a previous suggestion of fetal onset [16]. Our studies also show a lack of lymphatic vessels in the ovary before birth, although a substantial vascular network exists in the adjacent mesonephros and the extraovarian rete from late gestation (Fig. S1B, C). Therefore, we also analysed postnatal ovaries from the *Prox1*-EGFP transgenic mice to establish the onset of ovarian lymphangiogenesis.

**Figure 4:**
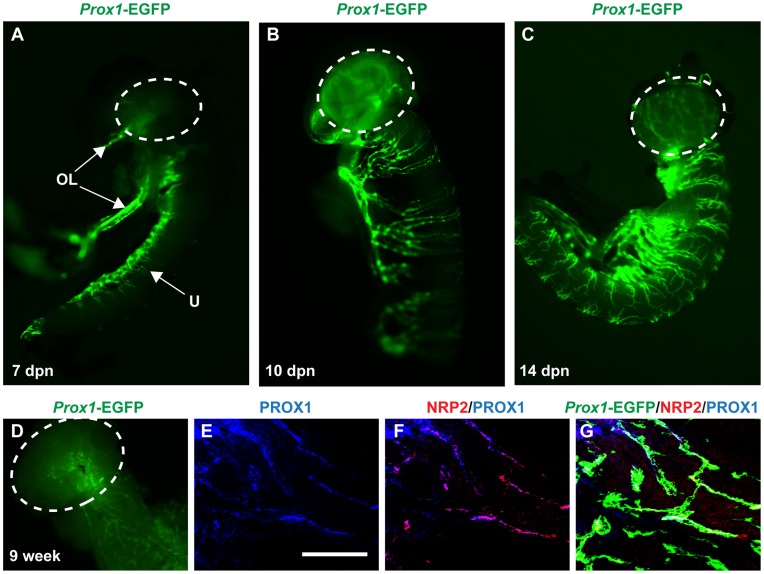
Lymphatic vessels develop in the postnatal ovary from around 10 dpn. A) At 7 dpn, EGFP-positive vessels are observed in along one side of the uterine horn and in the ovarian ligaments, but the ovary (encircled) is still devoid of lymphatics. **B)** At 10 dpn, the ovary possesses a lymphatic network. Lymphatic vessels sprouting laterally at distinct regional distances from a pre-existing vasculature along the length of the uterus have almost encircled the entire uterine horn. **C)** At 14 dpn, the ovary possesses a distinct lymphatic network and the uterine horn has developed a strikingly segmented lymphatic network encircling the entire tissue **D)** The adult ovary maintain a high Prox1-RGFP expression and the uterine horn has developed a extensive mesh of lymphatic vessels. **E)** Prominent *Prox1*-EGFP positive vessels of the 9 week uterine horn also express endogenous PROX1, and **F)** the lymphatic marker NRP2, **G)** both overlapping with *Prox1*-EGFP expression. OL = ovarian ligament; U = uterine horn; scale bar = 200 µm.

At 7 dpn, an extensive lymphatic network was present at one side of the uterine horn and along the pampiniform plexus, but not in the ovary proper (Fig. 4A). From 10 dpn, EGFP-positive vessels were observed in the ovary and lateral vessels were observed sprouting laterally at distinct sectional distances along the length of the uterine horn (Fig. 4B). At 14 dpn, the lymphatic network of the ovary became more distinct and the lymphatic vessels of the uterine horn encircled the entire structure (Fig. 4C). *Prox1*-EGFP expression is maintained into adulthood, but now with a much more dense network of vessels in the uterine horn (Fig. 4D).

To verify that the *Prox1*-EGFP transgene corresponds with endogenous PROX1 expression within lymphatic vessels, we performed additional whole-mount IF on female adult tissue. In the 9-week old uterine horn, endogenous PROX1 expression (Fig. 4E) co-localizes with the NRP2 (Fig. 4F) and *Prox1*-EGFP (Fig. 4G), as was previously shown for the male reproductive tract (Fig. 1). Hence, we are confident that *Prox1*-EGFP expression mimics endogenous PROX1 expression and demarcates the lymphatic vessels of the female reproductive organs.

### Adult Ovaries Possess an Extensive Lymphatic Network

**Figure 5:**
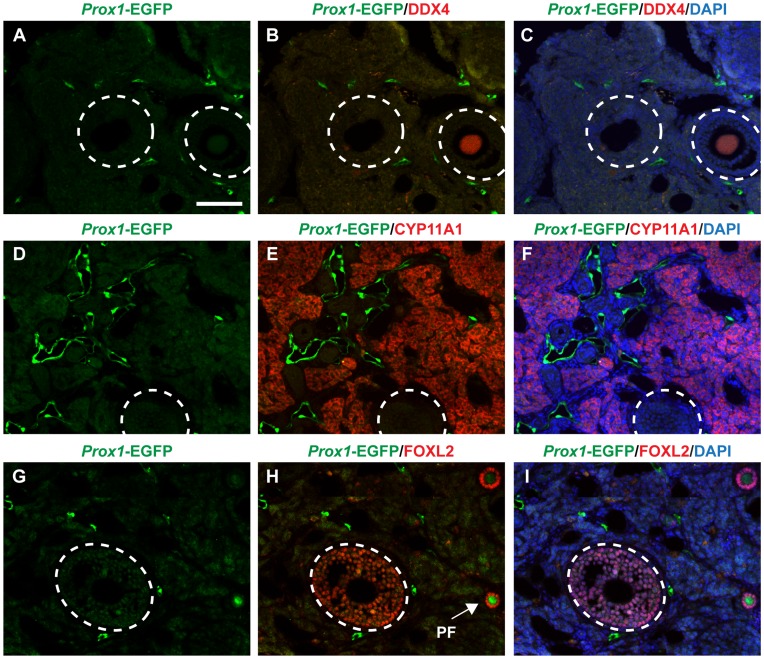
The adult ovary possesses an extensive lymphatic network. A–C) In the ovarian cortex, secondary follicles (encircled) with DDX4-positive oocytes are devoid of *Prox1*-EGFP positive lymphatic vessels, but lymphatic vessels are readily observed in close proximity. **D–F)** The ovarian medulla visualized by staining of the theca cell marker CYP11A1, contains a rich network of lymphatic vessels. **G–I)** The granulosa layer of secondary follicles localized by FOXL2-staining is devoid of lymphatic vessels, but with *Prox1*-EGFP positive vessels localizing very close to the border between the theca and granulosa layers (dotted circle). Oocytes of primary follicles containing a single layer of FOXL2-positive granulosa cells express a discernable level of *Prox1*-EGFP (arrow). Scale bar A = 100 µm.

To determine if internal EGFP-positive vessels exist in the adult ovary, we performed section IF on adult (9-week) *Prox1*-EGFP ovaries. Early stage follicles with DDX4 (DEAD box polypeptide 4)-positive oocytes were devoid of, but in close proximity to lymphatic vessels (Fig. 5A–C). In particular the ovarian medulla and the theca layer, as visualised by CYP11A1 (Cytochrome P450, family 11, subfamily a, polypeptide 1) expression contained a rich network of lymphatic vessels (Fig. 5D–F). On the other hand, no EGFP-positive vessels were observed in the granulosa layer of secondary follicles, as visualised by FOXL2 expression (Fig. 5G–I). Interestingly, oocytes of the primary follicles also showed *Prox1*-EGFP expression (Fig. 5H, arrow) which was downregulated as the follicles matured.

**Figure 6:**
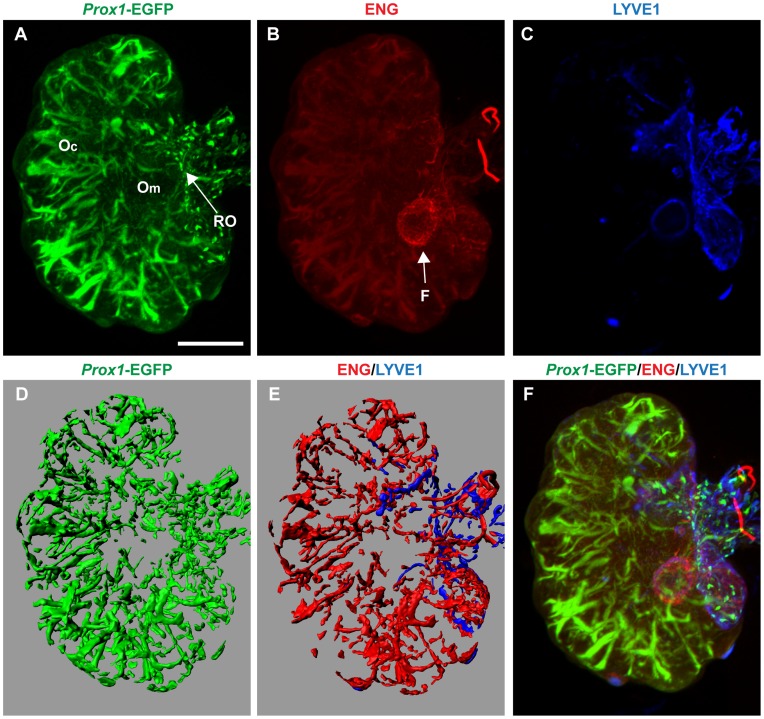
The adult ovary possesses a rich lymphatic network largely overlapping with the blood vasculature. Representative images captured from optical projection tomograph, also represented in Vid S2. **A)** The mature ovary contain a dense network of lymphatic vessels that emanate from the rete ovarii, and **B)** a rich network of ENG-positive blood vessels. **C)** Lymphatic vessels also expressing LYVE1 are generally localised to the ovarian and extraovarian rete. **D)** 3-D model of *Prox1*-EGFP positive lymphatic network as compared to **E)** LYVE1-positive lymphatic vessels and ENG-positive blood vessels **F)** Merged image of ENG, EGFP and LYVE1 expression in the adult ovary reveals distinct patterning of the blood and lymphatic network. Oc = ovarian cortex; Om = ovarian medulla; RO = rete ovarii; F = follicle; scale bar = 1 mm.

To fully appreciate the spatial distribution of the lymphatic network, we also performed whole-mount IF and OPT analyses on *Prox1*-EGFP adult ovaries. These experiments revealed a surprisingly extensive lymphatic network within the ovary proper (Fig. 6 and Video S2). The adult ovary possesses a rich network of ENG-positive blood vessels extending from the rete ovarii and into the cortex (Fig. 6B, E). In contrast to the developing testis, the ovary also contains an extensive lymphatic network within the ovary proper, with a patterning largely overlapping with the blood vasculature (Fig. 6A, D, F). The ovary also contains LYVE1-positive lymphatic capillaries that seem largely restricted to the rete ovarii (Fig. 6C, E, F).

## Discussion

Lymphatic vessels form in most organs, including testes and ovaries, but the spatial distribution of the network can vary both between tissues and species. Here, we present novel insights into the lymphatic vasculature of mouse gonads, based on high-resolution, three-dimensional visualization of the lymphatic network using a newly available and highly specific lymphatic marker strain of transgenic mice, *Prox1*-EGFP [28].

Over the last forty years, several studies have described the presence of lymphatics in testes and ovaries in different mammalian species [10], [11], [12], [15], [18], [19], [20], [21], [34], [35], [36], [37], [38], [39], [40]. Together, these studies point towards distinct lymphatic vascular morphologies between species in postnatal gonads. The early development of gonadal lymphatics has remained largely unexplored and the process and timing of initial lymphangiogenesis remains to be described. Further, in adult gonads, the lymphatic network has predominantly been visualized by LYVE1 expression and in two-dimensions. Here, we expand our knowledge by including additional molecular markers, predominantly PROX1, and also offer new structural insight by incorporating OPT analyses to describe the sexually dimorphic process of mouse gonad lymphangiogenesis. These studies also reveal an atypical formation of lymphatic vessels whereby collecting vessels are prevented from developing within the testis proper, instead being limited to the testis cap.

A previous study of blood vascularization of the fetal mouse gonads indicated that lymphangiogenesis was initiated in utero at around 17 dpc in both XY and XX embryos [16], but others have implicated the onset of ovarian lymphangiogenesis to the second week of postnatal life [21]. Our studies also show that lymphatic vessels appear on the testis cap at around 17.5 dpc, but that the fetal ovaries are devoid of lymphatic vessels. Although lymphatic vessels extend towards the ovary from late gestation, the ovaries themselves do not express lymphatic capillaries until 10 dpn, as previously suggested [21].

Hirai et al [18] recently showed that the testis interstitium in adult mouse testes is devoid of lymphatic capillaries, despite being characterized as a lymphatic space. Lymphatic vessels were only present in, and immediately subjacent to, the tunica albuginea, as observed by LYVE1 expression. We observed in the adult ovary a much more extensive network of PROX1-positive lymphatic vessels than LYVE1-positive vessels, but did not observe a similar subset of lymphatic vessels in the testis. Thus, our studies using the *Prox1*-EGFP marker corroborate the observations made by Hirai and co-workers. Combined, these studies add new insight into lymphangiogenesis of the fetal testis, showing how a rich lymphatic network first develops along the future spermatic cord and culminating in an abundant vascular plexus in the mesonephros adjacent to the rete testis. At later stages of gestation, new vessels are prompted to sprout across the testis cap, but somehow prevented to invade the testis interstitium.

This inhibition of lymphatic vascularization inside the testis proper may be specific to smaller rodent species and may not necessarily represent the lymphatic morphology of larger mammals. Nevertheless, the mere fact that lymphatic vessels form on the testis surface and inside the tunica albuginea ([18], and this study) is an intriguing observation that warrants further investigation. This morphological event suggests the presence of one or more inhibitory molecules, or alternatively lack of activation signals, in the mouse testis during late gestation and beyond. Therefore, the identification of these molecule(s) could prove valuable in the treatment of cancers by blocking lymphatic growth from malignant tumours, as a lymphatic network is intrinsic to immune cell trafficking and plays a major role in inflammation and tumour metastasis [3], [41].

Although it remains unclear what prevents lymphatic vessels from invading the testis, it is likely that the initial establishment of lymphatic network across the testis surface is, at least in part, hormonally regulated. Evidence for this comes from the adult ovary, where it has been shown that lymphatic remodelling in the cycling ovary is regulated by hormones intrinsic to the hypothalamic-pituitary-gonadal (HPG)-axis [21]. In XY mouse embryos, the HPG-axis appears to be activated at around 17 dpc [42], [43], which corresponds to the time when lymphatic vessels start to grow from the extratesticular rete and across the testis surface. Further to this, gonadotropic hormones have also been shown to regulate lymphangiogenesis during ovarian cancer [44].

Contrary to the testis, the adult ovary contains a rich network of internal lymphatic vessels, both within the medulla and also the cortex. Although follicles are devoid of lymphatics, the extent to which the cortical space is invaded by a mesh of larger lymphatic vessels was surprising (video S2). Previous studies using LYVE1 as lymphatic marker were not able to fully recognise this rich lymphatic network as these collecting vessels typically do not express LYVE1, but do express PROX1 [8], [25]. Therefore, as small lymphatic vessels typically express LYVE1, the lack of LYVE1-positive vessels throughout the ovary indicate that ovarian capillaries are either LYVE1-negative vessels, or that these PROX1-positive vessels represents larger collecting vessels that are folded in such a way that LYVE1-positive lymphatic capillaries distribute solely in the rete ovarii.

Clearly, significant sexual dimorphism exists in the timing of initial lymphangiogenesis of the gonads. In testes, lymphatic vessels first appear at around 17 dpc, 2–3 days prior to birth. In female mice however, lymphatic vessels do not invade the ovary proper until after birth at around 10 dpn. In the ovary, lymphatic growth has been linked to hormone signalling corresponding to folliculogenesis, also corresponding with an up-regulation of the pro-lymphatic genes *Vegfc*, *Vegfd* and *Vegfr3* [21]. It is well established that the process of blood vascularization of the gonads is sexually dimorphic [45]. Hence, it is also a possibility that the sexually dimorphic patterning of lymphatics is partly influenced by dimorphic signalling molecules regulating vascularisation, which occur earlier during development. In fact, VEGF signalling is hypothesised to play a instructive role in the different vascular patterning of testes and ovaries [46] and *Vegfa* has emerged as one factor involved in dimorphic blood vascularization [45], [47]. The dynamics between the various VEGF ligands and receptors that are at play during angio- and lymphangiogenesis remains a subject of intense studies both in development and disease. Thus, developing gonads represent a unique system to gain further insight into these processes.

## Supporting Information

Figure S1 **Testicular, but not ovarian lymphangiogenesis is initiated during late gestation in mice, as visualized with Prox1-EGFP transgenic gonad-mesonephros complexes. A)** Until 16.5 dpc, no EGFP-positive lymphatic vessels are observed in the developing testis, but are observed in the adjacent mesonephros from around 15.5 dpc. At 17.5 dpc a more extensive lymphatic network is visible along the spermatic cord and in the mesonephros, ultimately spanning out over the testis cap. From 18.5 dpc, Prox1-EGFP signal also appears from inside the testis proper, making visualization of superficial vessels difficult. T = testis; M = mesonephros. **B)** Comparable to XY development, the mesonephros of XX fetuses are observed to contain EGFP-positive vessels during fetal life, also prominent in the Müllerian duct between 15.5–16.5 dpc. No EGFP-positive lymphatic vessels are observed in the ovary proper during fetal life, but a rich vascular network is seen developing along the ovarian ligaments from 17.5 dpc. O = ovary; M = mesonephros; MD = Müllerian duct. C) At 19.5 dpc, no EGFP-positive vessels are observed inside the ovary, as visualized by section IF co-stained with the granulosa cell marker FOXL2. A prominent lymphatic vessel is detected in the extraovarian rete (arrow), and oocytes in the ovarian cortex are also expressing EGFP (aterisks). O = ovary; EoR = extraovarian rete; scale bar = 50 µm.
(TIF)

Video S1 **OPT reconstruction showing** ***Prox1*** **-EGFP localization in 17.5 dpc mouse testis as contrasted against ENG-positive blood vessels.** Whole-mount IF for EGFP and ENG was performed on *Prox1*-EGFP transgenic testis-mesonephros complexes. 3D-rendering show distinct organisation of the lymphatic network as it emanates from the spermatic cord to the rete testis, then sprouting out over the testis cap. No EGFP-positive lymphatic vessels are observable within the testis.
(M4V)

Video S2 **OPT reconstruction showing** ***Prox1*** **-EGFP localization in adult (9-week) mouse ovary contrasted against ENG-positive blood vessels and LYVE1-positive lymphatic capillaries.** Whole-mount IF for EGFP, ENG and LYVE1 was performed on *Prox1*-EGFP transgenic ovaries. 3D-rendering show distinct organisation of the lymphatic network as it emanates from the rete ovarii, a region also expressing LYVE1-positive capillaries, then into the medulla. An extensive network of *Prox1*-EGFP-positive, LYVE1-negative lymphatic vessels is observable throughout the ovary, including the cortex.
(M4V)
