# Supplementary figures and images for: Three-Dimensional Imaging of Prox1-EGFP Transgenic Mouse Gonads Reveals Divergent Modes of Lymphangiogenesis in the Testis and Ovary
Source: PLoS One. 2012 Dec 20;7(12):e52620. doi: 10.1371/journal.pone.0052620 (PMC3527586; doi:10.1371/journal.pone.0052620)

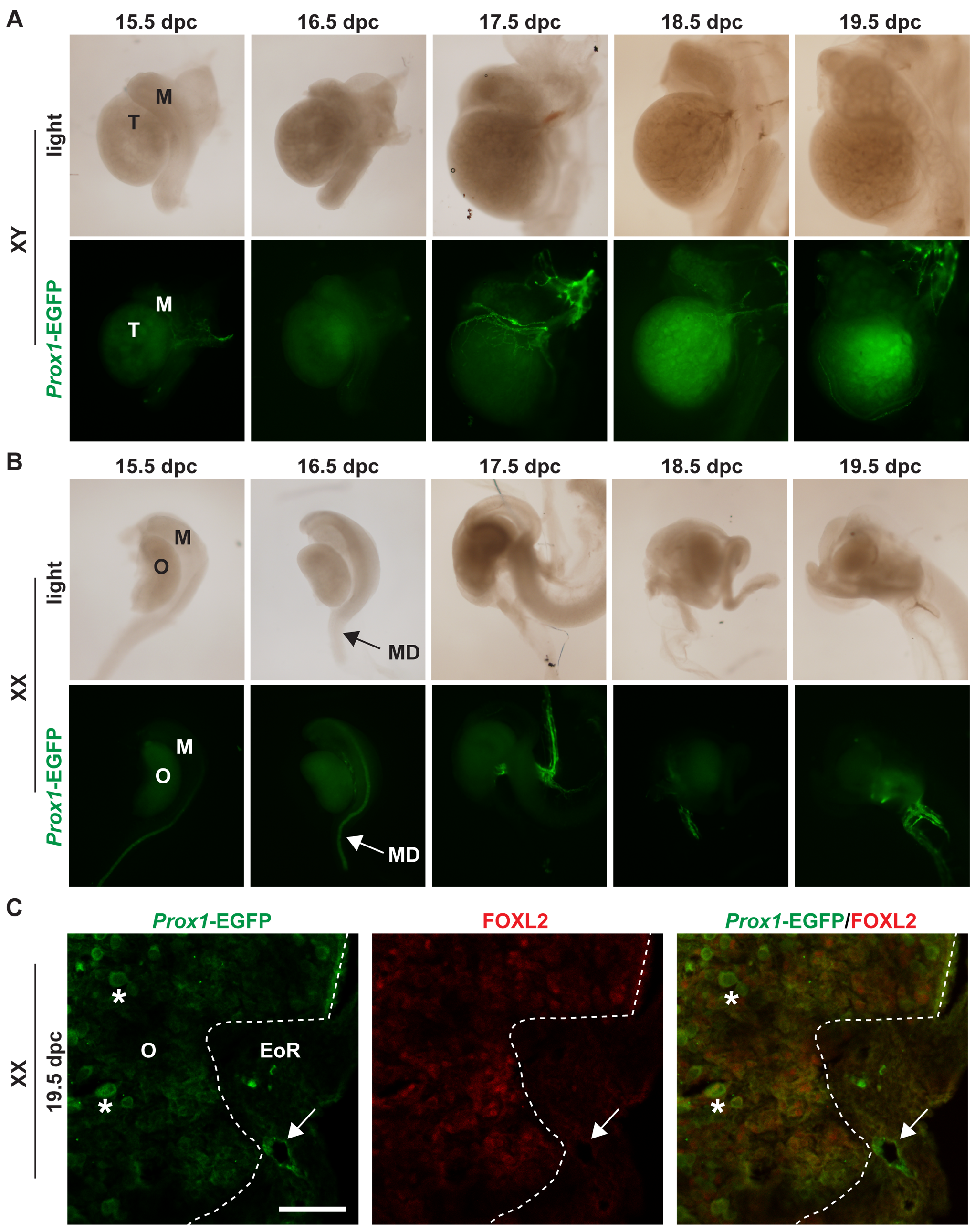

Supplement: Figure S1 — Testicular, but not ovarian lymphangiogenesis is initiated during late gestation in mice, as visualized with Prox1-EGFP transgenic gonad-mesonephros complexes. A) Until 16.5 dpc, no EGFP-positive lymphatic vessels are observed in the developing testis, but are observed in the adjacent mesonephros from around 15.5 dpc. At 17.5 dpc a more extensive lymphatic network is visible along the spermatic cord and in the mesonephros, ultimately spanning out over the testis cap. From 18.5 dpc, Prox1-EGFP signal also appears from inside the testis proper, making visualization of superficial vessels difficult. T = testis; M = mesonephros. B) Comparable to XY development, the mesonephros of XX fetuses are observed to contain EGFP-positive vessels during fetal life, also prominent in the Müllerian duct between 15.5–16.5 dpc. No EGFP-positive lymphatic vessels are observed in the ovary proper during fetal life, but a rich vascular network is seen developing along the ovarian ligaments from 17.5 dpc. O = ovary; M = mesonephros; MD = Müllerian duct. C) At 19.5 dpc, no EGFP-positive vessels are observed inside the ovary, as visualized by section IF co-stained with the granulosa cell marker FOXL2. A prominent lymphatic vessel is detected in the extraovarian rete (arrow), and oocytes in the ovarian cortex are also expressing EGFP (aterisks). O = ovary; EoR = extraovarian rete; scale bar = 50 µm. (TIF) [file pone.0052620.s001.tif]
